# The *Drosophila prage* Gene, Required for Maternal Transcript Destabilization in Embryos, Encodes a Predicted RNA Exonuclease

**DOI:** 10.1534/g3.116.028415

**Published:** 2016-04-07

**Authors:** Jun Cui, Yun Wei Lai, Caroline V. Sartain, Rebecca M. Zuckerman, Mariana F. Wolfner

**Affiliations:** Department of Molecular Biology and Genetics, Cornell University, Ithaca, New York 14853

**Keywords:** embryo, egg activation, mRNA stability, exonuclease, *Drosophila*

## Abstract

Egg activation, the transition of mature oocytes into developing embryos, is critical for the initiation of embryogenesis. This process is characterized by resumption of meiosis, changes in the egg’s coverings and by alterations in the transcriptome and proteome of the egg; all of these occur in the absence of new transcription. Activation of the egg is prompted by ionic changes in the cytoplasm (usually a rise in cytosolic calcium levels) that are triggered by fertilization in some animals and by mechanosensitive cues in others. The egg’s transcriptome is dramatically altered during the process, including by the removal of many maternal mRNAs that are not needed for embryogenesis. However, the mechanisms and regulators of this selective RNA degradation are not yet fully known. Forward genetic approaches in *Drosophila* have identified maternal-effect genes whose mutations prevent the transcriptome changes. One of these genes, *prage* (*prg*), was identified by Tadros *et al.* in a screen for mutants that fail to destabilize maternal transcripts. We identified the molecular nature of the *prg* gene through a combination of deficiency mapping, complementation analysis, and DNA sequencing of both extant *prg* mutant alleles. We find that *prg* encodes a ubiquitously expressed predicted exonuclease, consistent with its role in maternal mRNA destabilization during egg activation.

The transition from egg to embryo involves major changes in cell fate and potential, including progression of the cell cycle from meiotic arrest through completion of meiosis to the initiation of mitosis (reviewed in Clift and Schuh 2013; Horner and Wolfner 2008). This developmental transition involves major molecular changes in the egg including the polyadenylation of some maternal mRNAs (*e.g.*, for *Drosophila*: Benoit *et al.* 2008; Cui *et al.* 2008, 2013) and the degradation of others (*e.g.*, Chen *et al.* 2014; Giraldez *et al.* 2006; Kugler *et al.* 2013), synthesis of new proteins, and phospho-modulation of others (Guo *et al.* 2015; Krauchunas *et al.* 2012; Roux *et al.* 2006, 2008). The molecular changes of “egg activation” are critical for pronuclear formation and cell cycle modulation, for embryonic patterning and morphogenesis, and for structural and chemical changes to the egg’s outer coverings to block polyspermy and support the developing embryo.

Remarkably, most of this transition is driven or conducted entirely by parental (mostly maternal) molecules. In particular, maternally encoded mRNAs drive production of proteins needed for oocyte maturation and maintenance, for reversing this differentiated state after fertilization to permit totipotency, and for initiating early embryonic cell divisions and cell fate decisions. The maternal mRNAs needed for embryonic development must be kept stable until fertilization, and then must be translated at the appropriate time and place (*e.g.*, reviewed in Kugler and Lasko 2009; Tadros and Lipshitz 2009, 2005; Tadros *et al.* 2007b; Yartseva and Giraldez 2015). At the same time, for embryogenesis to proceed normally, maternal RNAs must be eliminated at the appropriate time and place (*e.g.*, Giraldez *et al.* 2006; Kugler *et al.* 2013) so that the zygotic genome can take over. For example, some cell cycle regulators must be eliminated in order for meiosis to resume (*e.g.*, Swan and Schüpbach 2007; Pesin and Orr-Weaver 2007) and complete, in preparation for pronuclear fusion and embryonic mitoses; indeed many of the maternally stored mRNAs that are degraded at egg activation have roles in cell cycle regulation, (*e.g.*, meiotic cyclins; Tadros *et al.* 2007a). Additionally, some mRNAs encoding localized proteins are initially present throughout the oocyte and undergo massive destabilization during egg activation except in protected local areas. Transcripts that fall into this latter category in *Drosophila* include those from the *Hsp83*, *nanos*, and *Pgc* genes (Bashirullah *et al.* 1999, 2001). Destruction of maternal mRNAs occurs in two general phases (reviewed in Laver *et al.* 2015). The first phase is maternally driven: products that had been loaded into the oocyte during oogenesis are activated, and they degrade certain RNAs; in *Drosophila*, 20% of stored maternal transcripts are subject to this degradation (Tadros and Lipshitz 2009). The second phase of degradation of maternal transcripts is dependent on zygotic gene expression. In *Drosophila*, an additional 15% of maternal transcripts are degraded under this control.

Identifying the regulators of stability and degradation of maternal RNAs has been challenging, both because egg activation is rapid and because many of its regulators are maternally encoded and therefore cannot be detected by looking for changes in the egg’s transcriptome. However, studies in model systems have identified some regulators of the fate of maternal mRNAs. For example, in *Drosophila*, the maternal phase of degradation requires activity of the PAN GU (PNG) kinase complex, causing translation and activation of another key component the SMAUG (SMG) protein (Tadros *et al.* 2007a). SMG binds to specific elements in certain maternal mRNAs, and targets these mRNAs for degradation by recruiting a deadenylase complex (Benoit *et al.* 2009; Chen *et al.* 2014; Semotok *et al.* 2008). In another example, a zygotically encoded miR small-RNA has been shown to mediate the degradation of maternal RNAs in zebrafish (Giraldez *et al.* 2006; Bazzini *et al.* 2012). A similar mechanism, with a different miR, likely operates in *Drosophila* (Bushati *et al.* 2008), and the piRNA pathway is also involved in regulating maternal mRNA stability in *Drosophila* embryos (Rouget *et al.* 2010). But knowledge of the machinery that selectively degrades maternal mRNAs is incomplete.

A genetic approach, such as that taken by Tadros *et al.* (2003) in *Drosophila melanogaster*, provides a way to identify important regulators of maternal mRNA stability. These authors identified several X-linked genes whose female sterile mutations affected the destabilization of maternally encoded *Hsp83* mRNA in early embryos (Tadros *et al.* 2003). Many of these loci were linked to key pathways during egg activation. Among the molecules identified in this screen was a conserved GLD2 poly(A) polymerase, *wispy*, which extends poly(A) tails of a large number of maternal mRNAs (Cui *et al.* 2008, 2013; Benoit *et al.* 2008), permitting their stability and, where tested, their translation (Benoit *et al.* 2008; Cui *et al.* 2008). Two loci were identified as *grauzone* and *cortex*, which were known to be required for completion of female meiosis (Page and Orr-Weaver 1996; Lieberfarb *et al.* 1996; Chen *et al.* 2000; Swan and Schupbach 2007). Genes encoding subunits of the early embryonic cell cycle regulator PNG kinase complex, including *png*, *plutonium (plu)*, and *giant nuclei (gnu)* (Lee *et al.* 2003), were also detected in the screen. Another mutation discovered in that screen was *prg*, whose molecular identity was unknown. Offspring from *prg* mutant mothers fail to destabilize maternal *Hsp83* mRNA, suggesting that PRG plays some role in maternal mRNA degradation. Here, we report molecular mapping and sequence analysis of *prg* mutant alleles that demonstrate that *prg* encodes a predicted RNA exonuclease, suggesting a role as part of the enzymatic machinery that degrades maternal mRNAs.

## Materials and Methods

### Drosophila stocks and complementation tests

*prg^16A^*/*FM6* and *prg^32^*/*FM6* (Tadros *et al.* 2003) were kindly provided by W. Tadros and H. Lipshitz (Hospital for Sick Children, University of Toronto, Canada). *Drosophila* strains carrying deficiencies [*Df(1)BSC719*/*Binsinscy*, *Df(1)ED6565*/*FM7h*, *Df(1)A94*/*FM6*, *Df(1)BSC530*/*Binsinscy*, *Df(1)260-1*/*FM4* and *Df(1)AD11*/*FM7c*] or *P*-element insertion in the *prg* region (P{MaeUAS.6.11}*CG42666^GG01337^*, *P{EPgy2}CG42666^EY21466^* and *P{XP}CG42666^d10828^*) were obtained from the Bloomington Stock Center. For complementation tests, we crossed approximately five 3-d-old virgin females of each strain to *prg^16A^* and to *prg^32^* males, and scored the fertility of their *prg*/*Df* (or P-insertion) female progeny.

### Nucleic acid extraction and PCRs

To identify the location of the mutant lesions in *prg*, whole fly genomic DNA was extracted from *prg^16A^* and *prg^32^* males as in Sirot *et al.* (2014) and used as template to amplify target regions using GoTaq PCR amplification kit (Promega, Madison, WI). DNA sequencing was performed by Cornell Life Sciences Core Laboratories Center (Cornell University, Ithaca, NY). To examine *prg* expression, total RNA was extracted from 3- to 5-d-old adult males, adult females, and embryos collected 0−2, 2−4, or 4−6 hr after egg laying, cDNA was synthesized, and RT-PCR carried out as described previously (Cui *et al.* 2008; Findlay *et al.* 2014). Primers for genomic and RT-PCRs are listed in Supplemental Material, Table S1.

### Data availability

The authors state that all data necessary for confirming the conclusions presented in the article are represented fully within the article.

## Results

### prage alleles carry nonsense mutations in CG42666, a gene that encodes a predicted exonuclease

**Figure 1:**
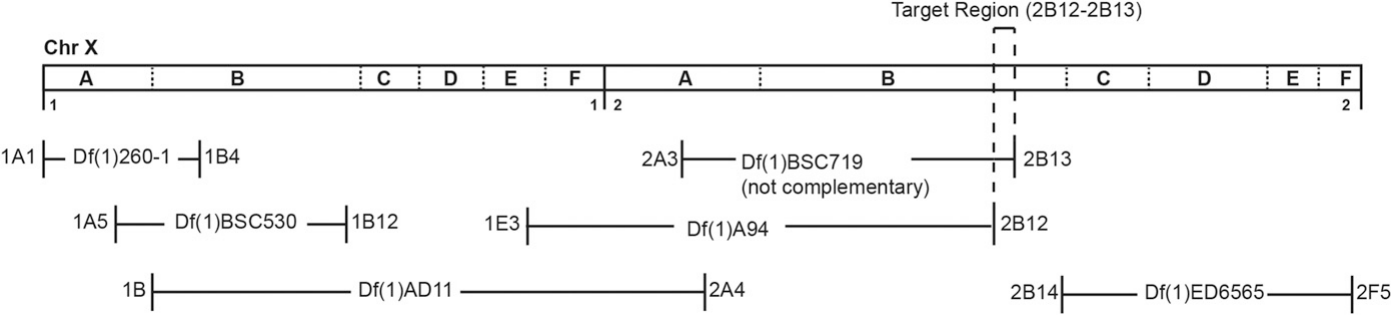
Map of the 1A-2F region of the X chromosome, showing location of the deficiencies used to narrow down the position of the *prg* gene.

**Figure 2:**
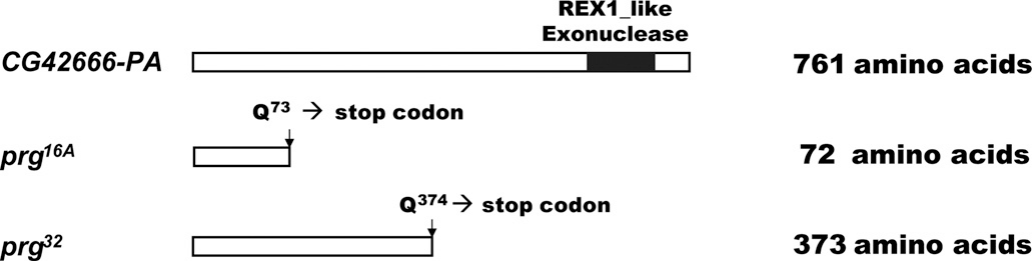
Schematic representation of mutant lesions in *prg* alleles. The cartoon shows a schematic of the PRG protein isoform PA, which encodes a protein of 761 amino acids. *prg^16A^* and *prg^32^* have nonsense mutations in the coding region that result in truncated proteins of 72 and 373 amino acids, respectively. The stop codons remove a similarly large C-terminal portion of all other PRG isoforms.

**Figure 3:**
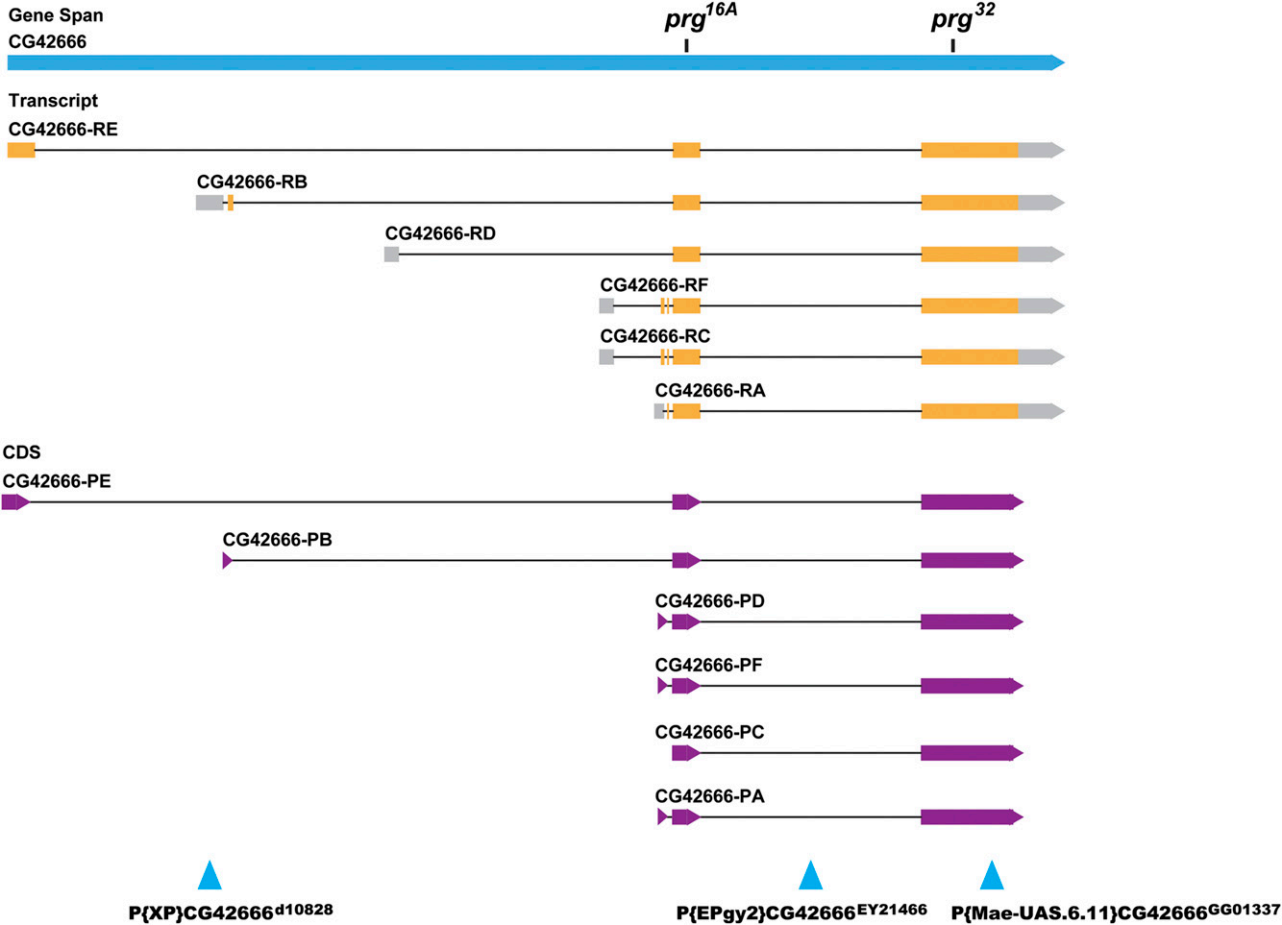
*prg* gene structure. The map shows the positions of the *prg* RNA and protein isoforms, and the locations of the *prg* mutant lesions, as well as the *P*-element insertions that were used in complementation tests to confirm gene assignment. Data are from Flybase (http://www.flybase.org). Orange denotes regions of the transcript that are translated into protein (purple shows those regions within the protein). Gray indicates exon regions that are not translated. Black bars mark the positions of the mutations in the two extant *prg* alleles. Blue triangles show the locations of *P*-element insertions.

The *prg* gene was previously reported as in polytene chromosome region 1B4-1E2 (Tadros *et al.* 2003). To localize the *prg* gene more precisely, we carried out complementation analysis between both *prg* mutant alleles (*prg^16A^* and *prg^32^*) and six deficiencies in or near the 1B4-1E2 region (Figure 1 and Table S2). *Df(1)BSC719* failed to complement both *prg* alleles, while another line, *Df(1)A94*, carrying a partially overlapping deficiency complemented both alleles. These results suggested that the *prg* mutation was in chromosome region 2B12-13. Genomic DNA corresponding to the predicted exons of four genes in this region [*CG14812*, *deltaCOP (CG14813)*, *CG14814*, *Med18 (CG14802)*] was PCR-amplified and then sequenced in both *prg* mutant lines. We found no difference from wild type in these four genes for either *prg* allele. Considering the possibility that the cytological breakpoint in the deficiency might not have been perfectly annotated relative to the genome sequence, we expanded our search to include three additional genes [*CG42666* (originally called *CG14801*), *CG14810*, *CG14811*] from the adjacent region, 2B10. No differences from wild-type sequence were seen in either *prg* mutant chromosome for *CG14810* and *CG14811*. However, as shown in Figure 2 and Figure 3 and detailed below, we found that both *prg* alleles have molecular lesions in the predicted ORF of *CG42666*. Each mutant contains a C-to-T single nucleotide change. In each allele this change generates a premature stop codon in the reading frame that results in a truncated protein. These data suggest that *CG42666* is the *prg* gene. Based on the *prg* mutant phenotype, one would expect *prg* RNA to be present in ovaries and early embryos. Our RT-PCR for *CG42666* RNA confirmed this expected expression pattern (Figure S1; see also Gelbart and Emmert 2013).

To confirm that the *prg* gene corresponds to CG42666, we carried out complementation tests of *prg* mutations with *P*-element insertions in *CG42666*. We tested for complementation between both *prg* mutant alleles and three *P*-element insertion lines available from Bloomington Stock Center. Two insertions, *P{Mae UAS.6.11}CG42666^GG01337^* (Mae) and *P{EPgy2}CG42666^EY21466^* (EPgy2), failed to complement both *prg* mutant alleles. However, *P{XP}CG42666^d10828^* (XP) unexpectedly complemented both *prg* alleles. We confirmed, by RT-PCR with primers specific to the XP line, that this line had an insertion in *CG42666* (Figure S2). Insertions Mae and EPyg2 are expected to disrupt all six RNA isoforms of *CG42666*, whereas insertion XP only interrupts the PE isoform (Figure 3). Our data suggest that disruption of this single isoform by the XP insertion does not eliminate function of *CG42666* gene; the other isoforms are likely expressed and produce functional PRG protein. Whether and how *CG42666* isoforms can compensate for each other requires further study, but the results from the Mae and EPgy2 insertion lines confirm that *CG42666* is the *prg* gene.

**Figure 4:**
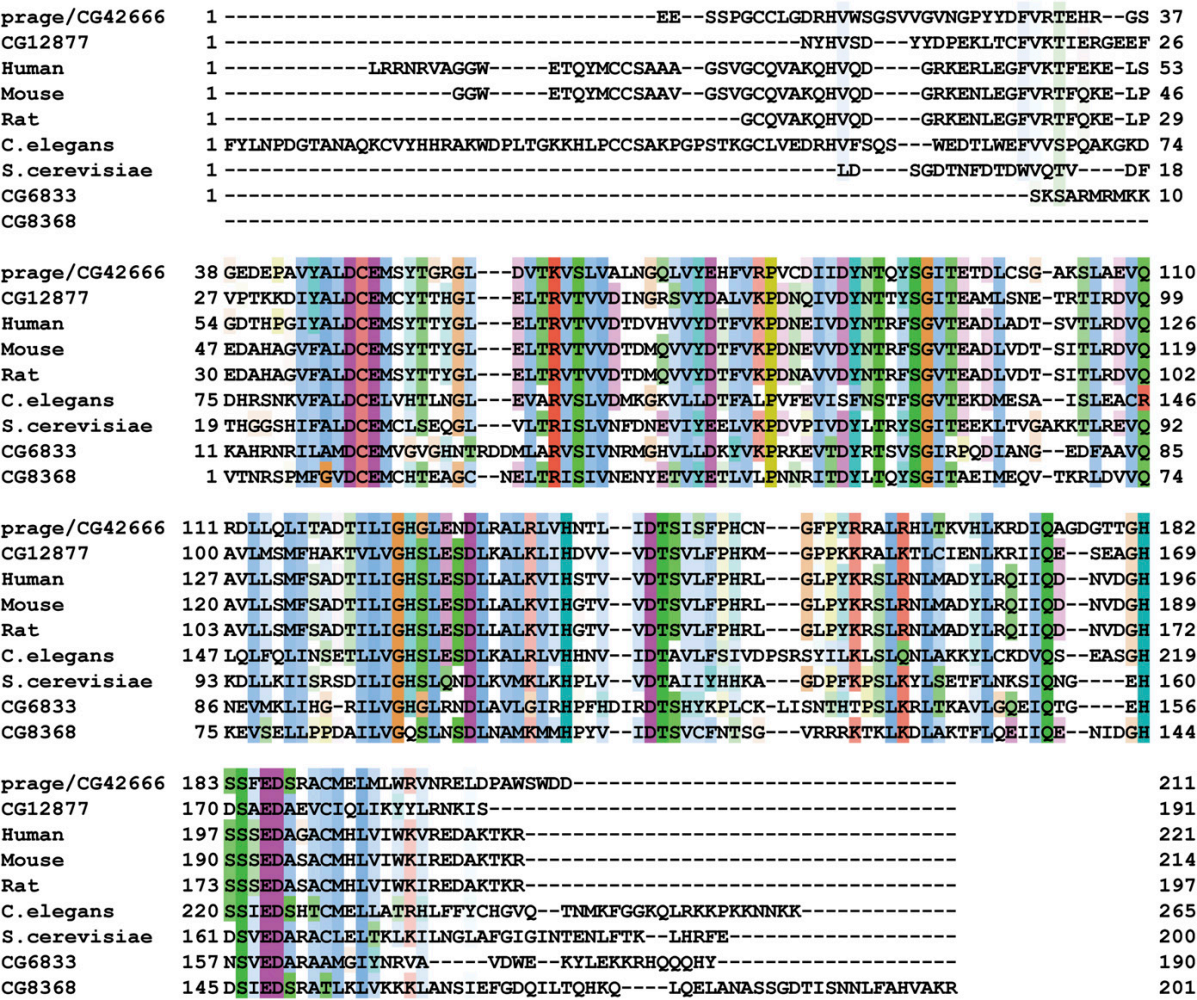
Alignment of REX_1-family genes. Amino acid sequence of the putative exonuclease domain of PRG was aligned against those of homologs from fly (CG12877, CG6833, and CG8368), human, mouse, rat, worm, and yeast. Identical or similar residues are highlighted in colored boxes.

*CG42666* encodes 6 RNA isoforms (PE, PB, PD, PF, PC, PA) with differing 5′ ends, according to the latest annotation of the *Drosophila* genome (http://www.flybase.org) (Figure 3). The PA isoform, for example, encodes a predicted protein of 761 amino acids. Sequence similarity analysis reveals that the CG42666 protein (hereafter called the PRG protein) is a putative RNA exonuclease: Interpro sequence analysis and classification identified a single conserved domain near the C-terminal end of the protein with terms “Exonuclease” (IPR006055), “Ribonuclease H-like domain” (IPR012337), and “Exonuclease, RNase T/DNA polymerase III” (IPR013520). Each of the mutant *prg* alleles has a single base pair change toward the 3′ end of *CG42666*, in the region that is shared by all six PRG isoforms. *prg^16A^* and *prg^32^* are both nonsense mutations, truncating their PRG proteins to 72 amino acids and 373 amino acids (relative to the PA isoform), respectively, and deleting the conserved exonuclease domain from each. Database searches revealed that the REX1_like exonuclease domain in the PRG protein is conserved among eukaryotes (Figure 4). In fruit flies, a domain of this type is also found in three additional genes. Of these three genes, the sequence of the predicted exonuclease domain of *CG12877* is the most similar to that of *prg*.

## Discussion

Egg activation is a coordinated process that is critical to initiate embryo development (reviewed in Clift and Schuh 2013; Horner and Wolfner 2008; Krauchunas and Wolfner 2013; Tadros and Lipshitz 2009; Tadros *et al.* 2007b; Yartseva and Giraldez 2015). Changes in the transcriptome during egg activation (Giraldez *et al.* 2006; Kugler *et al.* 2013) are fundamental, as they will allow changes in the spectrum of proteins in the cell that transition its state from that of differentiated mature oocyte to totipotent dividing embryo. One enzymatic player in transcriptome dynamics during *Drosophila* egg activation is known: the GLD2-family poly(A) polymerase encoded by *wispy* is essential for egg activation and early embryogenesis (Benoit *et al.* 2008; Cui *et al.* 2008). WISPY polyadenylates a large fraction of the maternally loaded mRNAs in the egg (Cui *et al.* 2013), presumably facilitating their efficient translation. But the machinery that catalyzes the degradation of maternal mRNAs during this transition is less fully understood. A genetic screen in *Drosophila* was successful in pinpointing candidates for roles in this degradation: genes whose mutants disrupted the destabilization of maternal mRNAs (Tadros *et al.* 2003). In this study we discovered that one of those genes, *prg*, encodes a predicted RNA exonuclease.

Approximately 55% of the *Drosophila* genome is represented as mRNA in the mature oocyte (Tadros *et al.* 2007b). Approximately 1600 (20%) of these maternally stored mRNAs are degraded upon egg activation. Tadros *et al.* (2007a) showed that two-thirds of these destabilized transcripts are regulated through the SMG protein, and are enriched for elements critical for cell cycle regulation. The remaining one-third are enriched for genes required for oogenesis (Tadros *et al.* 2007b). Evidence from yeast and *Xenopus* suggests that the first and often rate-limiting step in eukaryotic mRNA decay is the shortening of the poly(A) tail and the major deadenylase activity in *Drosophila* embryos is from the CCR4/POP2/NOT complex (reviewed in Temme *et al.* 2014). For two different mRNAs, SMG has been shown to recruit the CCR4/POP2/NOT deadenylase complex to the target mRNA, which shortens the poly(A) tail (Semotok *et al.* 2005; Zaessinger *et al.* 2006). Specific sequences in the 3′ UTR can target cytoplasmic mRNA for deadenylation, followed by either exosome (3′ to 5′) degradation or exonuclease (5′ to 3′) decapping/degradation (Houseley and Tollervey 2009). Both mechanisms require an exonucleolytic activity to complete the degradation (Houseley and Tollervey 2009). It is not known which exonuclease(s) degrade maternal mRNAs in *Drosophila* embryos.

The function of REX1-like proteins like PRG is unknown in most organisms. The only role that has been reported is in yeast; its *REXO1* gene’s function is required for RNA editing and maturation (Nariai *et al.* 2005; van Hoof *et al.* 2000). The sequence data presented here, in conjunction with the phenotypic data reported by Tadros *et al.* (2003) make it tempting to speculate that *prg* encodes an exonuclease that is actively involved in degrading maternal mRNA during the egg-to-embryo transition. Although it still remains to be demonstrated that the PRG protein has exonucleolytic activity, both *prg* alleles that fail to destabilize maternal mRNAs (Tadros *et al.* 2003) remove PRG’s predicted exonuclease domain.

PRG’s identity as a predicted RNA exonuclease raises several intriguing questions, beyond the obvious ones of its mechanism, potential partners, and targets. First, RNAseq (Gelbart and Emmert 2013) and microarray (Chintapalli *et al.* 2007) data indicate that the *prg* gene is expressed in stages and tissues that are unrelated to the egg-to-embryo transition (for example, it is expressed in adult males); we have verified some of these data (Figure S2). Although the existing *prg* mutant alleles remove its exonuclease domain and thus are likely null for this function in the germline (supported by the fact that homozygotes and hemizygotes are equally sterile), both are viable. This suggests that either *prg*’s activity is not needed in later somatic tissues, or that there are compensatory activities (perhaps from *CG12877* and/or the other two genes that encode proteins with exonuclease domains with some similarity to PRG’s). Alternatively, PRG’s translation might be regulated to restrict the protein’s presence to the female’s germline and early embryos. All of these will be fertile areas for future study.

Second, since some of the machinery required for the maternal/zygotic transition of the transcriptome is known, it will be intriguing to determine how *prg* relates to it. For example, the machinery includes the PNG kinase complex that upon egg activation triggers the translation of several maternal mRNAs including the one encoding SMG, the major factor that destabilizes maternal mRNAs in the early embryos (Tadros *et al.* 2007a). How does *prg* activity interface with the SMG-dependent pathway? Are *prg* and *smg* parts of independent pathways that act at different times? Or might *prg* control the stability of *smg* mRNA (assuming that *smg* mRNA must be translated upon egg activation), thus potentially regulating the amount of SMG or of components or assemblers of PNG kinase? Moreover, how is PRG itself regulated to act, including potentially to interface with the SMG pathway, so that it only degrades its targets after fertilization? Perhaps its targets are only modified appropriately at this time. Alternatively, perhaps PRG’s translation requires progression past a critical stage of early development, such as the meiotic progression mediated by the products of the *cortex* or *grauzone* genes (Lieberfarb *et al.* 1996; Pesin and Orr-Weaver 2007; Swan and Schüpbach 2007), or requires elongation of its poly(A) tail by the WISPY cytoplasmic poly(A) polymerase, as is the case for BCD (Cui *et al.* 2008; Benoit *et al.* 2008). It is also possible that PRG protein may be present in a nonfunctional state in oocytes, requiring post-translational modification during egg activation (*e.g.*, Krauchunas *et al.* 2012), or activation of a cofactor, or both, for its activity.

Finally, PolII binding assays (Chen *et al.* 2013) have identified *prg* as one of ∼100 genes that start significant transcription during cycles 8–12. That *prg* mRNA is both maternally loaded and also zygotically transcribed prior to the maternal-to-zygotic transition (MZT) raises the intriguing possibility that PRG could play roles in regulating RNA stability before the MZT (potentially even contributing to the initiation of the MZT), and also in the second wave of maternal mRNA degradation that occurs subsequently. Our identification of *prg* as *CG42666*, a predicted RNA exonuclease, permits the future investigation of these intriguing questions and further dissection of the molecular mechanisms that modulate maternal mRNA stability during the egg-to-embryo transition.

## Supplementary Material

Supplemental Material
