# Supplementary material for: The Drosophila prage Gene, Required for Maternal Transcript Destabilization in Embryos, Encodes a Predicted RNA Exonuclease
Source: G3 (Bethesda). 2016 Apr 7;6(6):1687–93. doi: 10.1534/g3.116.028415 (PMC4889664; doi:10.1534/g3.116.028415)
Supplement: Supplemental Material [file supp_6_6_1687__index.html]

The Drosophila prage Gene, Required for Maternal Transcript Destabilization in Embryos, Encodes a Predicted RNA Exonuclease — Supplemental Material 

# The *Drosophila prage* Gene, Required for Maternal Transcript Destabilization in Embryos, Encodes a Predicted RNA Exonuclease

## Supplemental Material for Cui *et al.*, 2016

**Files in this Data Supplement:**

- Figure S1 - *prg* transcripts are found in adults of both sexes and in embryos. (.pdf, 122 KB)
- Figure S2 - *P{XP}CG42666d10828* flies carry a P--element insertion at the expected position in the CG42666 gene. (.pdf, 151 KB)
- Table S1 - Primers used for genome sequencing to find the *prg* mutant lesions. (.pdf, 47 KB)
- Table S2 - Deficiencies tested to map *prage*. (.pdf, 87 KB)
