# Supplementary material for: The Drosophila prage Gene, Required for Maternal Transcript Destabilization in Embryos, Encodes a Predicted RNA Exonuclease
Source: G3 (Bethesda). 2016 Apr 7;6(6):1687–93. doi: 10.1534/g3.116.028415 (PMC4889664; doi:10.1534/g3.116.028415)

Figure S1. *prg* transcripts are found in adults of both sexes and in embryos.

RT-PCR was carried out on RNA extracted from 3-5d-old adult Oregon R P2 flies (Allis et al. 1977) or from embryos aged to the indicated times. *prg* RNA is detectable in all samples. *rp49* is used as a quantitation control. Primer sequences are available upon request.

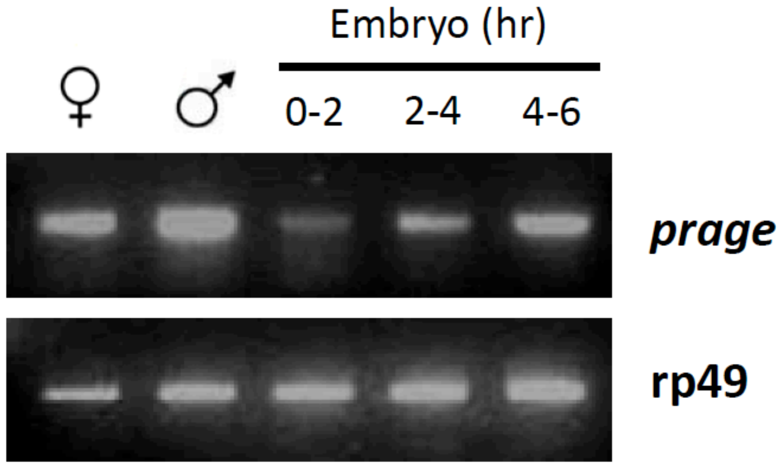

Supplement: Supplemental Material [file supp_g3.116.028415_FigureS1.pdf]
