# Supplementary material for: The Drosophila prage Gene, Required for Maternal Transcript Destabilization in Embryos, Encodes a Predicted RNA Exonuclease
Source: G3 (Bethesda). 2016 Apr 7;6(6):1687–93. doi: 10.1534/g3.116.028415 (PMC4889664; doi:10.1534/g3.116.028415)

Figure S2. ***P{XP}CG42666^{d10828}* flies carry a P-element insertion at the expected position in the CG42666 gene.**
Genomic DNA extracted from ten 3-4 day old adult male *P{XP}CG42666^{d10828}* flies was used for PCR analysis. *rp49* (lane 4) was used as an internal quantitation-control. Three different combinations of *prg* primers (sequences available upon request) shown in the cartoon give a PCR product of the expected size for CG42666 (primers P1+P2; lane 1) and P-element insertion in CG42666 (primers P1+P3; lane 2; and lack of band with primers P1+P4; lane 3). The size of the PCR product in lane 2 confirms that P-element P{XP}CG42666^{d10828} is located in the expected site. Lack of band in lane 3 is expected given the size of the P-element and the PCR extension times.

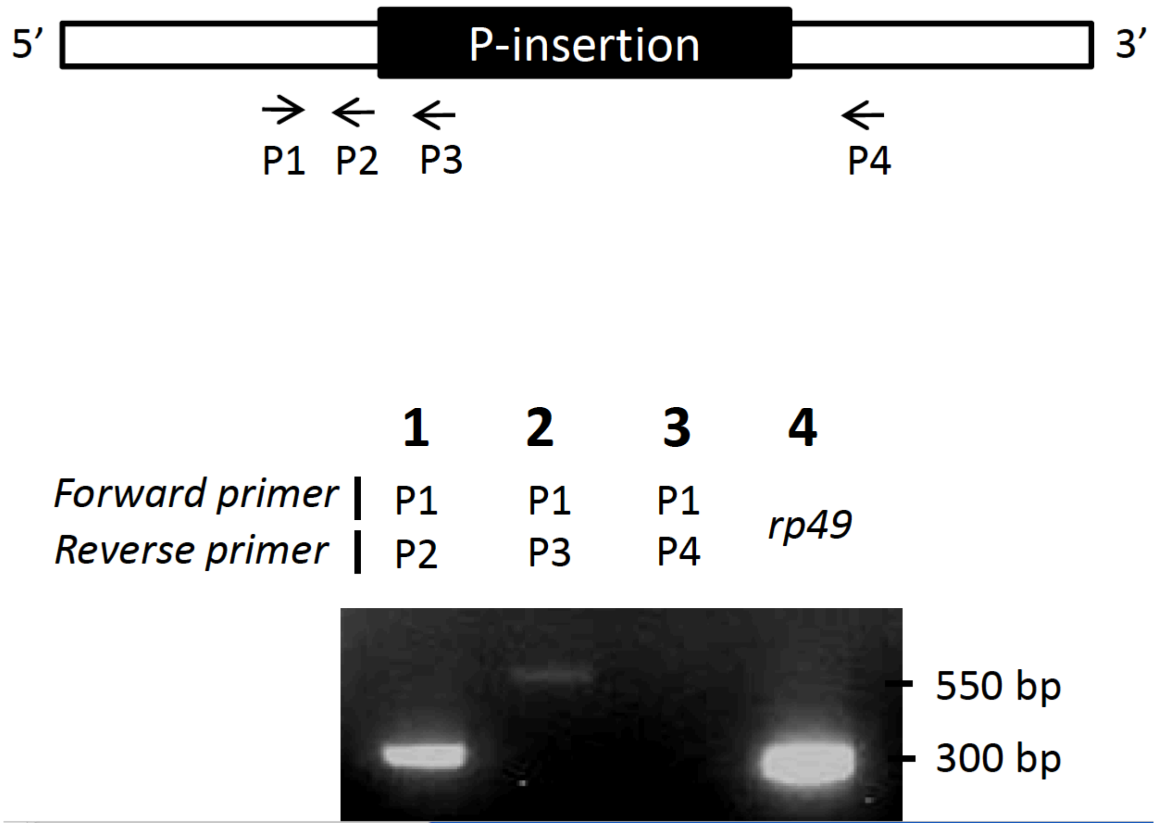

Supplement: Supplemental Material [file supp_g3.116.028415_FigureS2.pdf]
