# Supplementary material for: The Drosophila prage Gene, Required for Maternal Transcript Destabilization in Embryos, Encodes a Predicted RNA Exonuclease
Source: G3 (Bethesda). 2016 Apr 7;6(6):1687–93. doi: 10.1534/g3.116.028415 (PMC4889664; doi:10.1534/g3.116.028415)
Supplement: Supplemental Material [file supp_g3.116.028415_TableS1.pdf]

Table S1. Primers used for genome sequencing to find the *prg* mutant lesions.

Primer	Sequence, 5' -> 3'
prg-1	GTTGCACCTGCGCAAAAGAA
prg -2	TTCCCGCAAGCCAGGAGTTA
prg -3	CAGCGAAAGAGTCGGCAACA
prg -4	ACGTATGTGGGCGGACGACT
prg -5	GTTGGGAAATTTCAGCGATCA
prg -6	GCTGACGCCATCGTAGTACCG
prg -7	GCCGGTGGAAAGGAGTCTGTC
prg -8	GTCCTCCTCCTCCTTCGCTTG
