# Supplementary material for: The Drosophila prage Gene, Required for Maternal Transcript Destabilization in Embryos, Encodes a Predicted RNA Exonuclease
Source: G3 (Bethesda). 2016 Apr 7;6(6):1687–93. doi: 10.1534/g3.116.028415 (PMC4889664; doi:10.1534/g3.116.028415)
Supplement: Supplemental Material [file supp_g3.116.028415_TableS2.pdf]

Table S2. Deficiencies tested to map *prage.*

Deficiency	Deleted segment	*prg/Df* fertile*?
Df(1)260-1	1A1-1B4	Yes
Df(1)BSC530	1A5-1B12	Yes
Df(1)AD11	1B-2A4	Yes
Df(1)A94	1E3-2B12	Yes
Df(1)BSC719	2A3-2B13	No
Df(1)ED6565	2B14-2F5	Yes

*Fertility was tested with prg^{16A} and with prg^{32} alleles, for each deficiency.
